# Tobacco Industry Engagement in the House of Commons Science and Technology Select Committee E-Cigarettes Inquiry

**DOI:** 10.34172/ijhpm.8341

**Published:** 2024-08-28

**Authors:** Benjamin Hawkins

**Affiliations:** MRC Epidemiology Unit, Institute of Metabolic Science, Cambridge Biomedical Campus, Cambridge, UK.

**Keywords:** E-cigarettes, Tobacco Industry, Tobacco Control, Parliamentary Committees, Article 5.3, United Kingdom

## Abstract

**Background:**

A now extensive literature has documented political strategies of health-harming industries (HHIs), but little is known about their engagement with parliamentary select committees. Recent investments by trans-national tobacco corporations (TTCs) in electronic nicotine delivery systems (ENDS) has raised concerns that industry actors may be using these to re-engage policy-makers in ways precluded by the Framework Convention on Tobacco Control (FCTC) Article 5.3.

**Methods:**

This article examines tobacco industry engagement with the United Kingdom House of Commons Science and Technology Committee (STC) inquiry into e-cigarettes. It draws on a qualitative analysis of semi-structured interviews with committee members and support staff (n=4) triangulated against written and oral evidence submissions.

**Results:**

TTCs featured prominently in the STC inquiry via written and oral submissions. Opportunities existed for industry engagement, and potential influence, at each stage of the process. There was an absence of oral testimony from those sceptical about the potential health benefits of ENDS. The governance mechanisms in place for select committees appear inadequate for protecting committee work from industry influence. As it relates to TTCs, this has implications for the UK’s commitments under FCTC Article 5.3, yet understanding of the FCTC and the requirements of Article 5.3 compliance within the committee were limited.

**Conclusion:**

The governance of select committees requires urgent reform in order to balance norms of openness and participation with the need to protect their work from power of economic actors with conflicts of interest (COI). This is particularly the case in relation to TTCs and adherence to FCTC Article 5.3. These findings are of relevance to other select committees whose work affects the interests of HHIs. Further research is needed on other committees and sectors.

## Introduction

**Key Messages**

**Implications for policy makers**

- The governance and oversight mechanisms for parliamentary select committees should be revised in order to protect policy-making process from the influence of commercial vested interests.
- It should become established practise for all committee inquiries to appoint independent, external advisors through open and transparent procedures.
- Parliament (and other governmental agencies) should adopt clear policies on tobacco industry engagement and implement mandatory training for members and support staff in order to ensure compliance with the UK’s obligations under the World Health Organization’s (WHO’s) Framework Convention on Tobacco Control (FCTC).
- Committees should require all written submissions and those giving oral testimony to provide conflicts of interest (COI) statements in order for their evidence to be considered.
- To widen participation and ensure all relevant views are heard by committees, funding should be made available to facilitate testimony by under-resourced and geographically distant witnesses.

**Implications for the public**

Previous research has documented the extensive strategies of the tobacco industry to influence policy. The Framework Convention on Tobacco Control (FCTC) requires participating governments (including the UK) to ensure the tobacco industry cannot unduly influence health policy. Parliamentary select committee inquiries are an important part of the policy process. One such committee, The House of Commons Science and Technology Select Committee, held an inquiry into electronic cigarettes (e-cigarettes) in 2018. Many of these products are now owned by tobacco companies who participated extensively in the inquiry. This article examines the way the inquiry was conducted and identifies potential ways to protect committees from industry influence in future, to implement the FCTC, and deliver better policy. These include the appointments of expert advisors, attempts to identify conflicts of interests (COIs) in evidence submissions, and the provision of funding to allow a range of witnesses from different backgrounds to attend inquiry hearings.

A now extensive literature has documented political strategies of health-harming industries (HHIs) to influence policy-making,^1-7^ and to shape the evidentiary content of regulatory debates.^8,9^ HHI actors are highly pragmatic, well-resourced, and seek to engage decision-makers at all stages of the policy process, and in all relevant policy-making forums. Given the increasing importance of parliamentary select committees within the Westminster system,^10^ it would be out of keeping with the extensive evidence of their conduct in other areas of policy-making if HHI actors were not also seeking to influence committees’ work. To date, however, there have been only limited scholarly attention paid to this subject,^11^ and further study is needed to address this important gap in the literature. Moreover, attempts by HHIs to engage select committees and shape their outputs raise important questions about the governance mechanisms in place in relation to select committees and their engagement with commercial interests. These issues are particularly pertinent in relation to the global tobacco industry given the the United Kingdom’s undertakings as a party to the World Health Organization’s (WHO’s) Framework Convention on Tobacco Control (FCTC) Article 5.3 (from here on “Article 5.3”) to protect health policies from these vested interests.^12^

 As novel products, emerging only in the last decade, the evidence base about health effects of electronic nicotine delivery systems (ENDS)—such as electronic cigarettes (e-cigarettes) and heat-not-burn (HNB) devices—for users, and their wider impacts on population health, remains underdeveloped.^13-15^ Health researchers and advocates remain divided about the safety of these devices, their effectiveness in smoking cessation, the potentially negative externalities of their use for public health, and the correct regulatory regime which should apply to them.^16-19^ While the United Kingdom initially adopted a comparatively liberal regime on ENDS regulation, seeking to maximise access to the devices for smokers, the Government’s legislative agenda set out in the November 2023 King’s Speech included proposals to regulate the ENDS market and curtail youth vaping.^20^

 Policy debates on ENDS are further complicated by the significant investment of trans-national tobacco corporations (TTCs) within the sector.^21^ This has raised concerns that tobacco producers may be using these products to support their core cigarette businesses, and to circumvent existing tobacco control policies. ENDS, it has been argued, are the latest iteration of a decades-long tobacco industry strategy to use product innovation (eg, filtered and “low tar” cigarettes) to position themselves as part of the solution to tobacco-related harms with a legitimate role in the policy process.^22-24^ In keeping with this, TTCs have supported the “harm reduction” framing of ENDS policy as part of the wider appropriation of this concept by commercial actors to undermine evidence-based, population-level policies.^25^

 In this context, the 2017-2018 House of Commons Science and Technology Committee (STC) inquiry into e-cigarettes (and other ENDS) offers a potentially insightful case-study of how powerful economic actors may seek to engage select committees to further their policy agenda.^26^ Similarly, it provides an opportunity to examine the effectiveness of the measures in place to protect the work of select committees from the undue influence of tobacco industry actors as required by the FCTC.

 This article seeks to understand how the inquiry was instigated and conducted; how evidence was gathered, interpreted and synthesised; and how the focus of the report, its conclusions and recommendations were decided. In examining this process, it seeks to identify the opportunities for industry engagement, and the mechanisms put in place by the committee to insulate their work from conflicts of interest (COI) and to manage contacts with tobacco industry actors in light of Article 5.3. As such, the article generates insights of relevance beyond the current case, relating to wider issues of select committee governance, HHI engagement and Article 5.3 implementation across Parliament. The article concludes with recommendations about how the process of conducting inquiries on controversial issues, of relevance to HHIs, may be improved and how select committees can shield themselves from undue industry influence in line with the underlying norms of the FCTC.

## Background

 Research on the tobacco industry, and other HHIs, has identified the myriad ways in which well-resourced commercial actors seek to influence policy debates, engage decision-makers, and shape the regulation of their products.^3,27,28^ This includes engagement with, and co-option of, independent and legitimate sources of evidence, such as university-based scientists, think tanks and other institutions.^8^ Historically, this has occurred both directly and indirectly through industry-funded institutes and seemingly arms-length bodies and front groups.^29^ Since select committees are important sources of policy-relevant evidence, the cumulative prior knowledge of tobacco industry tactics suggests they will also seek to shape committee outputs.^11^

###  Product Development as Political Strategy

 The development of new “reduced-harm” products, such as filtered, low-tar and “light” variants, has been a key component of TTCs’ market and political strategies.^24^ Each of these were eventually proven to make no difference in safety.^30^ Despite this, investment in product innovation allows industry actors to position themselves as part of the solution to smoking-related harms and thus as key partners to government in the pursuit of public health. At the same time, the development of allegedly “safer” alternatives diverts attention from other tobacco control approaches and decentres the idea of smoking cessation, and preventing initiation, as policy objectives. The extensive investment by tobacco companies in ENDS can thus be seen as the latest iteration of this long-standing industry strategy.^21^

 After initial attempts by companies such as British American Tobacco (BAT) to develop and seek regulatory approval for licenced medical devices, the focus of the tobacco industry has shifted onto the marketing of ENDS as consumer products, targeted ostensibly at current smokers as a “safer” alternative to smoking conventional cigarettes.^31^ Different companies have focussed on different technologies in their product ranges. The most common are e-cigarettes, such as BAT’s *Vype *or Imperial Brands *Blu* products, in which a nicotine solution (often containing flavouring agents) is heated by a battery-operated coil or filament to produce an inhalable vapour. While Philip Morris International (PMI) and its sister company, Altria, have invested in the vaping sector – including the 2018 purchase of a 35% stake in the leading US brand *Juul*, from which it divested in 2023^32^ – its ENDS portfolio also includes HNB products such as its *IQOS* brand. Japan Tobacco International has also invested in both e-cigarettes (*Logic*) and HNB products (*Ploom*).

 The establishment of the PMI-funded *Foundation for a Smoke-free World*—and the prominence given to “reduced-harm” products on tobacco companies’ websites and corporate social responsibility materials—are explicitly designed to position TTCs as legitimate policy actors who are part of the solution to smoking-related mortality and morbidity.^33,34^ The diversification of TTCs’ product portfolios into ENDS has afforded them the pretext to seek engagement with policy-makers in ways precluded, or rendered politically problematic, by the FCTC. At the same time, it offers political cover to governments sceptical about the tobacco industry, or wary of reputational damage that could arise from such engagement.

###  Select Committee Governance

 Although they do not have formal responsibility for the scrutiny and adoption of legislation, House of Commons select committees play an important role in holding power to account through public hearings and inquiries.^10,11,35-38^ Previous analyses suggest that select committee reports can be influential in shaping government policy through their recommendations.^35,36^ Moreover, as select committee hearings receive increasing media attention, they have been identified as key sites in which the “drama” of the policy process is played out and enters the public consciousness.^38^ This can have a significant impact on how voters, and policy-makers who depend on their consent (or acquiescence), come to define and prioritise policy issues. Consequently, the work of select committees is of clear relevance to scholars seeking to understand the development of health and other public policy debates. Conventional accounts of select committees identify their independence as a key mechanism through which they are able to exercise their important oversight function, free from interference by government.^35^ However, far less attention has been paid to the governance and oversight of committees, and how they themselves should be held to account for their activities.^11^

 Select committees are established, and their remit and powers set out, by House of Commons Standing Order 152 including the ability to request information and evidence and to call (but not compel) persons to attend the committee. This places few obligations on committees in terms of governance and accountability mechanisms beyond a requirement to “report from time to time the minutes of evidence taken before sub­committees, and to lay upon the Table of the House the minutes of the proceedings of sub­committees” (Section 4c), and the ability of the house to remove committee members before the end of the parliament (Section 5). However, the specific circumstances in, and mechanisms through, which this would occur are not specified. In addition, while SO 152 foresees the desirability, and at times the need, for committees to appoint specialist advisors (Section 4c), no additional details are given on the process through which such advisors should be selected, and their specific role once in post.

 In light of this, committee members and, in particular, committee Chairs have significant freedom to identify issues of concern to them and undertake inquiries into these issues in the way they see fit. A key role in the design and execution of inquiries is also played by committee support staff, who undertake much of the design and organisation of inquiries and the drafting of reports and outputs. Given the wide-ranging focus of their inquiries, neither clerks nor even committee specialists are expected to be experts on all aspects of a committee’s work. Recognising this, committees may decide to appoint an external expert advisor—usually an eminent researcher or practitioner specialising in the topic in question—to support members and committee staff in conducting an inquiry, although there is no obligation to do so.

###  The Framework Convention on Tobacco Control

 The potential for investment in ENDS to enable TTCs to re-engage policy-makers became of particular relevance in the context of the FCTC, and the significant additional barriers this raised for governmental engagement with the tobacco industry in signatory states. Reflecting the significant evidence of tobacco industry efforts to shape policy, Article 5.3 places an obligation on signatory governments to take active measures to ward against the undue influence of the tobacco industry over health policy-making:

 “*In setting and implementing their public health policies with respect to tobacco control, Parties shall act to protect these policies from commercial and other vested interests of the tobacco industry in accordance with national law.”*

 The FCTC was supplemented in 2008 through the publication by WHO of guidelines^39^ on the implementation of Article 5.3. This included Recommendation 2.1, which states that:

 “*Parties [to the FCTC] should interact with the tobacco industry only when and to the extent strictly necessary to enable them to effectively regulate the tobacco industry and tobacco products.”*

 These guidelines take a broad interpretation of the remit and applicability of the Article, stating that:

 “*They also apply to persons, bodies or entities that contribute to, or could contribute to, the formulation, implementation, administration or enforcement of those policies.”*

 This includes:

 “*Any government branch (executive, legislative and judiciary) responsible for setting and implementing tobacco control policies and for protecting those policies against tobacco industry interests should be accountable.”*

 While the political acceptability of direct engagement with the tobacco industry has declined significantly in recent years, cross-country, comparative analyses of self-reported compliance measures adopted by parties to the agreement suggest that implementation of Article 5.3 remains sub-optimal and highly uneven both between and within polities.^40^ While in a global perspective, the United Kingdom ranks highly on Article 5.3 implementation in the Global Tobacco Interference Index,^41^ its implementation across government remains piecemeal and inconsistent. While individual departments and agencies, including the now defunct Public Health England, His Majesty’s Revenue and Customs, and the devolved administrations, have adopted guidelines on interactions with the tobacco industry, the approach taken to reducing tobacco industry interference is not systematic.^42^

 The emergence of ENDS, their promotion as potential smoking-cessation devices and the strategic investment in the sector by TTCs raises important questions in terms of Article 5.3 implementation and the definition of a tobacco industry actor. This includes the issue of whether tobacco companies (and their subsidiaries), otherwise excluded from health policy-making, should be considered legitimate stakeholders in their capacity as producers of ENDS. This has important implications not just for the governance of select committee inquiries, but the wider conduct of government.Article 5.3 refers to the activities of the tobacco industry to influence tobacco control policy. Given the obvious relevance of ENDS for wider issues of tobacco control and the inextricable linkages between TTCs’ electronic and combustible businesses, the activities of tobacco companies in the guise of ENDS producers fall within the remit of Article 5.3.

## Methods

 The article draws on semi-structured interviews triangulated with documentary analysis of the committee report, written evidence submissions and transcriptions of oral testimony provided to the inquiry (Brinkman 2014). All committee members (n = 11) and support staff (n = 5) during the 2017-2019 parliament—a total of 16 potential respondents—were approached for interview via email and telephone. A total of 4 STC committee members and support staff (ie, clerks and committee specialists) who worked on the inquiry were interviewed. The remaining 12 either did not reply to, or declined, the interview requests, giving a participation rate of 25%. In addition, background discussions were held with scientists who have served as expert advisors to health-related select committees (n = 3), but these are not cited below. The interviews were conducted between January and April 2022 and lasted for between 35 and 75 minutes using the interview protocol supplied in the supplementary materials. All interviews were recorded on an encrypted device, transcribed, and stored along with recordings on a secure remote drive on university servers.

 Interviews were analysed qualitatively following the procedure for thematic coding set out by Braun and Clarke,^43^ with coding undertaken manually by the author in Microsoft Word. Transcripts were initially read to identify key themes and issues. Transcripts were then re-read, and examples of the identified themes were highlighted in the text. All relevant sections of the transcripts were then extracted, collated in a single document, and organised into a coherent order. This formed the basis of the analysis presented in the results section. Due to the small number of potential respondents, and the highly contentious nature of the topic, all participants have been fully anonymised and all quotations and other references to the interview data have been presented without attribution to protect respondents’ identities.

 The committee report was examined to identify the sources of evidence (ie, written submissions or oral testimony referenced to support its findings and recommendations). Written submissions were accessed via the committee inquiry web page and categorised as to their provenance (ie, whether they were from the independent e-cigarette industry, TTCs, independent vaping companies, government agencies, health service providers, civil society organisations, etc). These categorisations were then aggregated into wider groupings (eg, government, health, research, industry) to give a clearer picture of the arena from which respondents emerged. Witnesses invited to give oral testimony before the committee were similarly categorised. Written evidence submitted by the four main TTCs active in the UK ENDS market – BAT, PMI (via its UK subsidiary Philip Morris Ltd), Japan Tobacco International, and Imperial Brands – were downloaded and analysed to reconstruct industry actors’ policy positions on ENDS, and the rhetorical strategies they employed to promote their preferred regulatory model and to reintegrate themselves in the health-policy architecture.

 Given the emphasis placed by interviewees on oral evidence (see below), witness testimony before the committee was examined in detail. All oral evidence sessions before the committee were watched by the author twice via Parliament TV and notes taken on any aspects of the hearings that related to the primary research questions on committee governance, tobacco industry influence, and the application of Article 5.3. The data collected in this phase constitutes an asynchronous, non-participant observation study of the committee hearings. Note was taken of any COI statements from oral witnesses relating to engagement with TTCs, or other relevant industries. Finally, transcriptions of oral evidence sessions involving these companies and a tobacco industry trade association were downloaded from Hansard – the official transcript of parliamentary proceedings – and subjected to a discourse analysis alongside industry written submissions using the same methodology employed for the interviews described above. To clarify the terminology used when reporting the findings below, those giving oral evidence before the committee are referred to as “witnesses,” while those interviewed for the study are referred to as “respondents” and quotations are attributed to interview respondents by number to maintain anonymity. Ethical approval for the study was granted by the ethics board of the Unit to School of Humanities and Social Science at the University of Cambridge (approval number: 21/286).

## Results

 The Table below presents evidence submissions to the inquiry by disaggregated and aggregated sectors. This presents different levels of detail about respondents and the fields from which written submissions and witness testimony emerge. Where sectors cannot be aggregated into a wider category (eg, members of the public, think tanks) these are presented as separate categories in both the disaggregated and aggregated columns. The STC received 95 written submissions, plus 7 supplementary submissions providing additional information or clarifications. As interview respondents commented, this volume of evidence was very high compared to other inquiries run by the STC. Of the 95 initial submissions, 20% (n = 19) came from the “nicotine” industry, comprised of the tobacco industry-owned (n = 7) and independent (n = 12) ENDS producers. This was the same number as from the aggregated health sector (n = 19). The research community provided 17% of submissions (n = 16). A further 13 submissions came from the pharmaceutical and other industry actors, while 11 came from e-cigarette user groups and 9 from national, regional and local government actors. Together these groups represented over 90% of the submissions received.

**Table:** Evidence Submission to the Science and Technology Committee E-Cigarettes Inquiry by Sector

**Disaggregated Sector**	**Written Evidence Submissions**^a^	**Oral Testimony**^b^	**Aggregated Sector**	**Aggregated Submissions**	**Aggregated Oral Testimony**
Government (national, local devolved)	4	2	Government	9	7
Regulatory and government agencies	4	5			
Parliamentarians	1	0			
Health associations, charities, and NGOs	15	2	Health	19	4
Smoking cessations and other health service providers	4	2			
Research organisations (eg, universities)	9	0	Research	16	7
Individual researchers	7	7			
Independent e-cigarette industry	12	2	Nicotine industry	19	6
Tobacco industry	7	4			
Pharmaceutical industry	3	0	Other industries	13	0
Miscellaneous industries	7	0			
Industry consultancies	3	0			
ENDS user groups	11	1	ENDS user groups	11	1
Think tanks	2	0	Think tanks	2	0
Private citizens	6	0	Private citizens	6	0
**Total**	**95**	**25**	**Total**	**95**	**25**

Abbreviations: NGOs, non-governmental organizations; ENDS, electronic nicotine delivery systems. Source: Written submissions/details of oral evidence sessions published on the STC e-cigarettes inquiry website. ^a^ Where individual submissions are authored by more than one individual from the same sector they are counted as a single submission. Supplementary submissions are not counted, except for the Medicines and Healthcare Products Regulatory Agency and PHE. As they provided individual supplementary evidence of similar depth to their original joint submission, they are considered to have contributed independently. ^b^ Each witness giving oral evidence is counted individually.

 In terms of oral evidence, a total of 25 individuals gave oral testimony in committee hearings. Of these, 28% (n = 7) were government actors and a further 28% were researchers. A total of 4 witnesses were from TTCs and 1 was from a trade association representing TTC members. This meant 20% (n = 5) of all oral witnesses came from the tobacco industry. In addition, a sixth industry witness represented an “independent” ENDS manufacturers’ trade association (ie, those not associated with the tobacco industry). Of the 7 academic researchers appearing as witnesses, 6 were psychologists or behavioural scientists studying tobacco use and addition. The seventh was a Professor of Internal Medicine, Riccardo Polosa, who had previously received tobacco industry funding.^44^ All respondents providing written submissions and the oral witnesses attending each evidence session are listed in the committee report. The inquiry did not appoint an expert advisor.

###  The Power of Oral Evidence

 All interviewees and background informants agreed that oral evidence is particularly impactful on committee members and thus in shaping the direction of the inquiry, its report, and recommendations. The e-cigarettes report contained 47 references to oral evidence provided to the committee compared with 53 references to written submissions received. This was despite the fact that there were only 25 witnesses who appeared before the committee versus 102 primary (95) and supplementary (7) written submissions. However, simple quantitative metrics like this miss the important qualitative differences between written and oral evidence such as the theatrical or performative elements of oral evidence sessions identified in previous studies.^38^ These may have a particular impact on committee members and support staff. For example, in evidence Session 1, on January 9, 2017, Prof. Polosa held up some pieces of burnt toast to underline the similar toxicity of e-cigarette vapour. The unexpected appearance of the toast drew an audible reaction from both the committee and public gallery.

 Interviewees agreed that testimony from people individually impacted by an issue or policy are particularly powerful for committee members. As one respondent commented, oral testimony can “bring a subject to life” (Respondent 1). A second respondent concurred:

 “*Is there a different value to the evidence? Absolutely there is in that the evidence that you hear orally always carries more weight in a sense into what goes into the report” *(Respondent 3).

 In addition, committee members receive a link to written evidence of oral witnesses in the preparation materials they are supplied with for the evidence sessions. As such, getting selected to testify before the committee may be doubly beneficial as it also promotes witnesses’ written evidence (Respondent 1).

 Another input to committee inquiries are field visits to sites of relevance to the inquiry focus such as a factory or laboratory. Two respondents identified that these can be very impactful on committee members (Respondent 1, 3). The effect(iveness) of such events is similar to that of witness testimony in that they create a sense of immediacy, personalise abstract policy debates, and relate them to the lives and interest of specific individuals working in, or depending on, a particular facility. Resource-rich policy actors, such as businesses with relevant sites to visit, thus enjoy an advantage over other actors in seeking to influence the focus and outputs of committee inquiries. For examples, in their written submissions, Philip Morris Ltd offered to organise such a trip to their laboratories. In addition, one respondent reported that industry actors had offered to organise an event for the committee in which they could try vaping products (Respondent 3).

 There was evidence also that policy advocates were aware of the power of oral evidence to shape the inquiry’s findings. A number of actors contacted the committee lobbying to be included in the oral evidence sessions and explaining the importance of the particular insights they sought to bring. As one respondent commented:

 “*A lot of organisations tend to kind of make themselves known and say that you know, ‘we are going to submit written evidence but we are also very keen to come’ and, or, ‘we have a specific take on the lived experience within this area and we would really like to connect you with some people who could provide that input’”* (Respondent 2).

 The respondent was clear that anyone contacting the committee has “equal access” but this ignores the issue of capacity and resources needed to take the time to testify, the types of actors viewed as experts, as well as questions of power which impact the likelihood of being listened to.

 There was evidence that tobacco industry actors were able to shape the direction and focus of the inquiry via their written and oral submissions. For example, the different technological focus of the companies in terms of their ENDS products—with some companies focussed on e-cigarettes and others on HNB—meant time was devoted to these issues which could otherwise have been spent on other aspects of the debate. As one respondent commented:

 “*When we hit the sort of tobacco producers […] one side were heating it, one side was the vaping of it […] that monopolised a little bit of the inquiry, which with hindsight was not a waste of time, but was a distraction from the overall [topic]. And I think the effect of that was that the work that we could do on the flavourings and indeed the attraction that it would be to young people, which some groups were raising at the time, took up less of the report than actually, if we look at where we are now, it perhaps should have done” (*Respondent 3).

 The ability of tobacco companies to focus on the intra-industry cleavages in technology, as each company sought to promote the most favourable framing of their specific products, diverted attention from important health and regulatory considerations of relevance to the entire ENDS category. This included consideration of the strategic role of ENDS within tobacco companies’ overall business model. As the previous respondent continued:

 “*That’s where the difficulty with the tobacco companies came in because obviously although [they] were very quick to say they had no intention of widening their smoking market, clearly what they were trying to do was provide an alternative to smokers and I think nobody on the committee really believed that, […] but we didn’t explore that aspect of it. We didn’t explore the concept of a growing market by shifting away because […] we were looking at it from a cessation point of view”* (Respondent 3).

 This demonstrates not just how the inquiry’s initial terms of reference can lead it to focus on a highly circumscribed understanding of the issue at hand, but the ability of tobacco companies to shape an inquiry in often subtle ways, diverting attention from key issues and towards their favoured issue framings.

###  Openness, Inclusivity and Balance

 According to respondents, the key principles underpinning the evidence gathering phase of the inquiry were openness and inclusivity in order to hear a balanced range of views. Paraphrasing one respondent, this was in order to hear as many perspectives as possible on different aspects of the topic (Respondent 2, 4). Moreover, there was an awareness that if certain actors were omitted from evidence sessions, it may draw criticism from excluded groups—particularly those, such as industry actors, with sufficient resources to raise grievances—or be seen as delegitimising the inquiry. As one respondent commented:

 “*I don’t think [the committee] wanted to be in a position where the report was undermined afterwards by or picked apart by others saying ‘well but you didn’t even hear from X, Y and Z’” *(Respondent 1).

 However, ensuring that the committee heard a range of perspectives on certain issues proved difficult. As one respondent commented:

 “*There were certain areas we were really struggling to find people with a level of expertise to do it and to find people who could counter the arguments that we were getting in the written papers”* (Respondent 3).

 Respondents flagged the lack of resources available to committees to enable respondents to attend oral sessions as a particular problem (Respondent 1, 3). As one interviewee commented:

 “*It became a game of who can we get to come to parliament to give evidence [...] when you look at the academics and the researchers, they tend to be London, golden triangle [ie, Oxford, Cambridge and London] based […] obviously the tobacco companies at the drop of a hat would offer to come along” *(Respondent 3).

 Similarly, it was difficult to call international witnesses before the committee:

 “*We wanted people from America, we were trying to get evidence about why they were banning stuff but it didn’t happen and obviously that then didn’t follow through into the report” *(Respondent 3).

 Oral testimony thus tends to come from those in close geographical proximity to parliament and those actors such as large corporations, who have the personnel and economic resources to attend committee hearings. For example, of the three witnesses based overseas, two were from the tobacco industry.

 Despite the stated commitment to openness and balance, there was a notable absence of public health researchers and advocates from the oral evidence sessions, who might have articulated a more precautionary approach to ENDS. While the ENDS debate in the United Kingdom indeed had, to this point at least, focussed principally on their efficacy as smoking cessation devices, this framing of the issue was out of keeping with the approach taken in most policy contexts including, as the respondent cited above states, the United States.^45^ Despite the limitations on calling witnesses from overseas, there were UK-based researchers able to articulate this precautionary perspective. This included those who had submitted written evidence to the committee but did not appear as witnesses.

###  Tobacco Industry Engagement & Article 5.3

 Respondents reported that there had been very little discussion about the potentially negative effects, and wider ethical implications, of inviting tobacco industry actors to give oral testimony before the committee, even in their capacity as ENDS manufacturers and marketers. Asked if this had been a point of contention for the committee, one interviewee responded:

 “*There wasn’t really […] much discussion in private about the fact that tobacco companies owned many of the players in the vaping industry. […] I’m not of the sort of mindset where you, to coin a phrase, ‘cancel’ evidence because of where it’s come from. You should of course seek to avoid being naïve about interests and, you know, understand how you should interpret evidence from a particular source given their past behaviour or their economic interest or whatever it might be. But […] I don’t find it attractive the idea of saying, we’re not going to listen to you because you come from tobacco. You know, you should not be giving them a sort of excessive role, but you should hear the arguments that are put” *(Respondent 4).

 This was reflected by another respondent:

 “*I don’t remember it being enormously controversial the question of whether someone from the tobacco industry was in front of the committee. Of course, it’s up to the committee then to decide how they weight and interpret what a witness says” *(Respondent 1).

 A third respondent reported that the issue of the tobacco industry was discussed, demonstrating an awareness amongst the committee about their controversial history:

 “*We had a sort of 25, 30 minute discussion about the fact that obviously the tobacco industry had historically hidden the dangers of smoking […]. But at no stage did we come to an even really contemplative view of not hearing from the tobacco companies, but we did so very much on the basis that we wanted to hear what they say, rather than welcoming them as the solution to the problem that we’d identified”* (Respondent 3).

 However, instead of excluding TTCs, the aim was to find opposing viewpoints that would counter tobacco industry actors’ perspectives, in keeping with the norms of openness and balance discussed above:

 “*We were trying to find a counterbalance to that evidence, so the clerks spend a lot, spent a lot of time about trying to find recognisable experts that could counter the discussion that the tobacco companies would invariably give because we’d seen from their evidence papers where they were going to”* (Respondent 3).

 Yet, as noted above, there was an absence of voices dissenting from the industry-favoured, harm-reduction framing within the oral evidence sessions.

 The issue of tobacco industry engagement was raised by Prof. Jamie Brown in the Oral Evidence Session 1 (9 January 2018), and he was reassured by the Chair, Stephen Metcalfe, that it was possible for the committee to meet with the industry in their capacity as researcher producers:

 “*Dr Brown: What rules are you governed by in consulting the tobacco industry?*

####  Stephen Metcalfe: If they have conducted research, I believe we are entitled to invite them here and ask them, as long as it is a scientist as opposed to the PR people.” 

 The inquiry report does not contain any references to Article 5.3 or discussion of any potential COI of oral witnesses and those providing written evidence. The first oral evidence session did not ask for COI statements at the outset, though one committee member asked the academics testifying for this halfway through. Following this intervention, the second part of the session with other researchers did begin with COI declarations. However, this practise was not continued consistently throughout the evidence sessions when other researchers and civil society actors gave evidence. This reflects an inadequate engagement with the issue of COI by the committee and the lack of formal mechanisms to manage this. As one respondent commented, committee staff did some “due diligence” on oral witnesses but not written submissions (Respondent 3), the majority of which did not include COI statements.

## Discussion

 The analysis presented here builds on previous studies of the corporate political strategy of the tobacco industry,^3,28^ and HHIs more generally in the United Kingdom and gloablly.^1,4^ It focuses on select committees as a neglected component of the policy process within this literature. In so doing, it builds on previous studies of select committee governance,^11,35^ civil society participation in select committee inquiries^46^ and the performative impact of committee activities on policy-making.^38^ It identifies a number of concerns about the functioning and governance of select committees and their engagement with the tobacco industry or other commercial actors, which will be of interest to scholars beyond the immediate field of tobacco control and the UK policy context.

 At each stage of the process, there are opportunities for commercial actors to engage the committee and to attempt to shape its work. In most instances it is perfectly legitimate for them to do so. Yet such engagement needs to be properly governed in a way which guarantees a genuine plurality of inputs into committee work. While committees may operate on principles of openness and inclusivity, this can ignore the* de facto *ability of certain actors to shape both processes and outputs. In keeping with previous studies, respondents, noted that resource imbalances between actors influence who is able to participate in select committee inquiries.^47^ This extends beyond just the financial means to attend hearings to the ability to monitor activities, engage consistently, and to demand interests are taken into account. Powerful economic actors, such as TTCs, are able to engage at all stages of the inquiry process, in pursuit of their interests, with respondents indicating this can influence the design and conduct of inquiries. Consequently, written responses and witnesses appearing before the committee may not fully reflect the state of the research literature and the degree of consensus between scholars in the field.

 In the e-cigarette inquiry, the perspectives of health or policy actors based outside the UK did not form a significant part of the oral evidence feeding into the committee’s inquiry. The absence of international perspectives is important since e-cigarette policy debates in other contexts have differed greatly in tone and content from those in the United Kingdom.^14,45,48^ While this may have been a question of logistics, it is less clear why there were no UK-based public health researchers appearing before the committee to articulate the precautionary approach to ENDS which predominates in other policy contexts. In contrast, there was a disproportionate representation of tobacco industry actors and other witnesses emphasising the harm-reduction potential of ENDS who did appear before the inquiry.

 In highly contested policy contexts such as this, the norms of openness and inclusivity need to be supplemented with a concern for representativeness in order to ensure that the full range of legitimate, scholarly, and practise-related positions on a topic are represented within the evidence presented to the committee. This requires careful evaluation of written responses and planning of oral evidence sessions by committee staff. Since committee staff, by their own admission, cannot be expected to be subject experts on all aspects of a committee’s work, the appointment of and external expert advisor to oversee an inquiry may have helped address these issues.

 The extent of tobacco industry participation in the inquiry (representing 20% of all oral witnesses) has relevance also for the UK’s implementation of the FCTC.^40^ The role of tobacco companies in the production of e-cigarettes appears to be highly relevant to the way in which they were perceived by the committee. This had both ethical and practical components. In terms of the former, the fact that TTCs were producing potentially harm-reducing products seemed to assuage concerns that the committee may have had about previous industry activities. In terms of the latter, technological expertise possessed by TTCs, for example in the production of nicotine at scale, meant they were able to position themselves as key actors in any attempt to reduce smoking-related harms. As discussed above, this created a platform from which they were able to engage, and seek to influence, the focus of the STC inquiry and wider policy debates.

 The analysis above suggests that, while committee members had some knowledge of the sensitivities involved in engaging with industry actors, there was no specific consideration of Article 5.3 in the execution of the inquiry, or limiting the participation of TTCs in oral evidence sessions. This is, however, not an isolated example and is indicative instead of a more general issue with Article 5.3 compliance across government.^42^

 The findings here raise a wider set of questions about tobacco industry engagement in policy-making and legislative processes in their guise as ENDS producers, and the interpretation and implementation of Article 5.3 by Parliamentarians seeking to regulate this evolving sector. The analysis above will be of relevance to scholars, tobacco control advocates, and health policy-makers in other policy contexts.

###  Implications for Policy

 The analysis presented above suggests a number of changes which could be made to the governance of select committees and the conduct of their inquiries. *First*, there is a clear need for awareness-raising about issues of corporate political strategy, COI and, in dealing with the tobacco industry, the UK’s obligations under Article 5.3 within parliament. This could take the form of compulsory training for MPs and committee support staff*. Second*, in light of Article 5.3 and the particularly influential nature of oral evidence, tobacco industry actors should not be afforded disproportionate access to, and undue influence over, select committee hearings. Parliament should follow other government agencies such as Public Health England, His Majesty’s Revenue and Customs, and the Welsh Government in developing clear guidelines on tobacco industry engagement in the context of select committees and other capacities, in line with WHO guidance.

 The specific issues related to the tobacco industry speak to a wider set of concerns about commercial influence over select committees and the opportunities for corporate capture afforded by the currently weak committee governance and oversight mechanisms. A *third* recommendation is, therefore, that it should be mandatory for written submissions to a select committee and those giving oral evidence to provide COI statements. Committee support staff should take proactive steps to verify these and identify omissions for all evidence informing committee outputs and recommendations. *Fourth*, given the key role that committee support staff and Chairs have in setting the parameters of inquiries, evidence gathering and synthesis, and reporting they should be provided with additional training in research methods and scientific practise to facilitate their work. *Fifth*, and relatedly, the work of committees should be supported by expert advisors appointed to all inquiries on the basis of clear and transparent appointment procedures. *Finally*, given the importance of oral evidence in enacting the drama of the policy process,^46^ the disproportionate ability of well-resourced commercial actors to attend oral evidence sessions,^49^ and wider issues about the representativeness of select committee witnesses, funds should be available to facilitate the testimony of all relevant actors regardless of means, through the reimbursement of travel, accommodation and subsistence costs.

###  Strengths and Limitations

 This study builds on the previous analyses of select committees by critically examining their own governance structures and accountability mechanisms. In so doing, it addresses a clear gap in the literature in this area. In addition, it adds to our understanding of the political strategies of the tobacco industry, and the wider commercial determinants of health at a point in time in which TTCs are seeking to shape their regulatory environment and engage policy-makers through diversification into new product categories. This has important implications for the implementation of Article 5.3 and the governance of select committee inquiries related to tobacco and other HHIs (eg, alcohol and hyper-processed foods).

 However, the article has some limitations. First, it is impossible to say, on the basis of the data presented, the extent to which TTCs were able to influence the committee’s findings and recommendations, just that mechanisms and opportunities existed for TTCs to engage the committee. Second, this is a case study of a single inquiry from a single committee and, as such, the generalisability of the findings is unclear. Further studies into the work of the STC, and other committees whose work touches on the interests of HHIs, are needed to expand the insights generated here on both committee governance and industry influencing strategies. Third, the number of respondents was lower than hoped for. This likely reflects both the demands on parliamentarians in the aftermath of Covid, the fact that some MPS had left the committee or parliament, and the controversial nature of the policy topic. At 25%, the response rate is not untypical for qualitative research of this kind but, given the small pool of potential respondents, the overall number of formal interviews was limited to four. However, this meant that those who did participate had direct first-hand experience of the inquiry and were thus well placed to discuss its conduct and outputs. Furthermore, these accounts were supplemented through triangulation with additional data sources. Finally, it was impossible to ascertain from publicly available information or interviews the number of potential oral witnesses approached by the committee to give evidence but who were unable or unwilling to attend.

## Conclusion

 This article examines the engagement of TTCs in the conduct of a STC inquiry on ENDS. While recognising the importance of committee autonomy in their oversight function and holding government to account, it highlights the need for better governance of select committee evidence gathering, synthesis, and production processes. This is particularly important in the context of increasing awareness about the corporate political strategies of HHIs and the UK’s obligations under the FCTC. The highly politicised nature of ENDS policy, the contested nature of the evidence-base within public health, and the clear commercial interests of the tobacco industry make this a “paradigm case” of where vested interests may seek to subvert the activities of a select committee. Consequently, it is a clear instance of why effective governance responses to this threat may be required to ensure the integrity of select committees’ work and their role within the wider policy process. The article proposes recommendations, which could be adopted by the STC and other committees to improve governance practise and their potential impact on policy debates. While it focuses on a single committee inquiry, it raises wider questions about the work of parliamentary committees and the influencing strategies of other industries which should form the basis of further inquiry.

## Acknowledgements

 I would like to thank Prof. Martin White for supporting this work.

## Ethical issues

 This study was approved by the MRC Epidemiology Unit Ethics Committee, University of Cambridge, UK.

## Conflict of interests

 Author declares that he has no conflict of interests.
